# A Practice Survey of Canadian Forensic Sexual Behavior Programs

**DOI:** 10.1177/10790632251377706

**Published:** 2025-09-11

**Authors:** Payton McPhee, Jordyn Monaghan, Skye Stephens

**Affiliations:** 1 Department of Psychology, 3427 University of New Brunswick, Fredericton, NB, Canada; 2 Department of Psychology, 7991 York University, Toronto, ON, Canada; 3 Department of Psychology, 3690 Saint Mary’s University, Halifax, NS, Canada

**Keywords:** sex offenses, risk assessment, sexual offender treatment, prevention, forensic sexual behavior programs

## Abstract

Forensic sexual behavior programs provide assessment and treatment services to individuals who have committed a sexual offense and/or who are at risk of sexually offending. In Canada, practices vary across these programs as publicly funded community-based forensic sexual behavior programs do not adhere to a federal standard of practice. Although several practice surveys have been developed in previous years to explore techniques within these programs, updated research is needed due to recent developments in the field. The present study provides a comprehensive overview of assessment, treatment, and preventive practices in Canada by surveying 16 publicly funded Canadian forensic sexual behavior programs. Results found that programs generally followed evidence-based practices when conducting risk assessments, although adherence to evidence-based guidelines showed greater variation for treatment. Additionally, 70.6% of surveyed programs reported providing prevention services to individuals who have not crossed a legal sexual boundary, although approaches to these services varied across programs. Findings from the present study allow for a stronger understanding of forensic sexual behavior program practices nationwide and have implications for clinical practice.

## Introduction

In Canada, forensic sexual behavior programs provide services to those who sexually offend or are at risk of doing so. These programs often focus on tertiary prevention that involves service provision to individuals after they have sexually offended with the aim of preventing recidivism (Krug et al., 2002; MacDonald, 2002; Miele et al., 2023; Seto et al., 2024). Within tertiary prevention, there is strong support for the Risk-Need-Responsivity (RNR) model (e.g., Bonta & Andrews, 2017; Hanson et al., 2009). When translated into practice, the RNR model highlights the importance of conducting comprehensive risk assessments to identify salient risk factors to be addressed via intervention that is responsive to each client’s learning style (Bonta & Andrews, 2017). Additionally, the Good Lives Model (GLM; Ward & Stewart, 2003; Ward & Gannon, 2006) represents a strengths-based approach that can be integrated with RNR which aims to reduce the risk of recidivism by identifying individuals’ strengths and building upon their capacity to live a fulfilling and meaningful life (Ward & Brown, 2004). However, this is a more recent approach that is less extensively supported in the literature (e.g., Andrews et al., 2011). In contrast, secondary prevention involves service provision to those who are at risk of sexual offending and/or have no official criminal history (MacDonald, 2002; Seto et al., 2024). Secondary prevention is less developed, and evidence-based practice has not been formally established (e.g., Beier et al., 2009, 2015; Lätth et al., 2022; Seto et al., 2024; Stephens et al., 2022).

Generally, individuals who have been charged with a sexual offense are often mandated by the judicial system to attend a forensic sexual behavior program, where they undergo a risk assessment to inform sentencing and participate in treatment (Subramonian & Severn, 2020; Williams, 1995). Specialized forensic sexual behavior programming operates across community, correctional, and private sectors (Ellerby et al., 2010). Community and correctional programs are supported via federal and provincial funding and do not typically involve direct costs to clients (McGrath et al., 2010). This is in sharp contrast to the funding structure of forensic sexual behavior programs in the United States, where 90% of clients pay for forensic sexual behavior programs (Ellerby et al., 2010; McGrath et al., 2010).

Funding for specialized forensic sexual behavior programs is dependent on program accreditation in some countries (e.g., Scotland), which ensures that services conform to evidence-based practice (Aarten et al., 2011; Rex et al., 2003). While accreditation standards were adopted within Canadian federal correctional programming (i.e., within Correctional Services Canada), this is not reflected in all community-based programs (Hanson & Yates, 2004). With 11.1% of community-based adult forensic sexual behavior programs in Canada utilizing external consultations for quality improvement, funding allocation may be influenced by factors such as policy priorities and public demand rather than evidence-based practices (Jung et al., 2020; McGrath et al., 2010). Overall, this suggests that service provision and program-specific practices likely vary across the country, with limited research to understand the current state of practice within forensic sexual behavior programs. Practice surveys are useful to explore how these programs operate and offer guidance to support improvements in service delivery essential for evidence-based risk assessments, treatment outcomes, and preventive services.

### Findings from Previous Practice Surveys

Several notable practice surveys have provided an overview of practices in the field. McGrath et al. (2010) conducted a comprehensive practice survey of forensic sexual behavior programs in Canada and the United States. Their practice survey focused on programs that provide risk assessment and treatment services to adults, adolescents, and children (*N* = 549 surveys representing 1379 programs, 80% based in the community). In total, there were 34 completed surveys from Canada, representing 72 distinct programs (the remainder of the programs were from the United States). Results for risk assessment practices found that the Static-99 (Hanson & Thornton, 1999) was the most commonly used actuarial tool in Canadian community-based treatment settings with adult male clients (68.4%). The STABLE-2007 and ACUTE-2007 (Hanson et al., 2007) were the most used dynamic tools for this population (57.9%). Dynamic risk instruments were found to be incorporated more frequently into risk assessments in Canada (63.3%) compared with the United States (48.5%). McGrath et al. (2010) also found that few Canadian programs measured risk for general recidivism in conjunction with sexual recidivism risk. In addition, sexual preference testing was commonly incorporated into assessments in Canada and the United States, with 57.9% of adult male community-based programs using penile plethysmography, Viewing Time, or both as a tool(s) to measure sexual preference. According to McGrath et al. (2010), Viewing Time was more commonly used in the United States (45.8%) compared to Canada (10.5%), whereas phallometric testing was more frequently used in Canada (36.8%) compared to the United States (27.9%).

In addition to assessment, McGrath et al. (2010) surveyed programs on their treatment practices. All programs in Canada offered group-based treatment, but individual treatment was also frequently offered (94.4%). There was variation between the United States and Canada regarding treatment dosage. Community-based treatment within American adult programs spanned a median of 24 months, whereas Canadian adult programs spanned eight months. When asked to identify their primary treatment model within group therapy, Canadian programs indicated that the most frequently used model was cognitive behavior therapy (CBT; 47.4%), followed by relapse prevention (15.8%), and the Good Lives Model (10.5%). Notably, Risk-Need-Responsivity was only selected as the primary treatment model by 5.3% of participating Canadian programs that provided treatment to adult males in the community. In addition, treatment targets were sometimes at odds with what the literature deems as effective. For example, offense responsibility (78.9%) and victim empathy (89.5%) were cited as treatment targets by participating Canadian programs, despite little evidence-based support for the effectiveness of these targets in reducing offending rates (Hanson & Morton-Bourgon, 2005; Mann & Barnett, 2013; Mann et al., 2010).

Two subsequent practice surveys are relevant to highlight. In a practice survey exploring Canadian correctional institutions (federal, provincial, and territorial agencies; 14 jurisdictions), Bourgon et al. (2017) examined general risk assessment practices among 20 professionals who provided assessments within their jurisdiction. Most surveyed practitioners noted the use of sexual recidivism assessment tools for individuals who have committed a sexual offense (12/14 jurisdictions reported routine use of these instruments). Similar to McGrath et al. (2010), the Static-99R (Phenix et al., 2016) was the most used risk instrument (81.8%), followed by the STABLE-2007 (72.7%), and the ACUTE-2007 (45.5%). A major emphasis by Bourgon et al. (2017) was the considerable variation in risk categories/levels across instruments, suggesting that clients’ risk levels are managed differently depending on the risk tool used in each jurisdiction.

Finally, Kelley et al. (2018) conducted a practice survey in the United States that focused on risk assessment practices among American evaluators (*N* = 119) who worked with individuals who have sexually offended. Results mirrored previous research in that the Static-99R prevailed as the most routinely used static tool. Researchers categorized actuarial static risk instruments as old or new and found that most practitioners (84.9%) relied on newer instruments. Many evaluators (58.0%) reported only using one static tool when assessing risk and routinely included a dynamic instrument (most frequently the STABLE-2007, 42.0%). Of note, just over 20% of participants reported not routinely using an instrument to assess dynamic risk. There was less frequent inclusion of measures focused on protective factors (10.1%), with the most frequently used instrument being the Structured Assessment of Protective Factors for Violence Risk (SAPROF, a tool that focuses on protective factors for violence; de Vogel et al., 2012).

### Present Study

Despite the existence of previous practice surveys that have examined assessment and treatment with those who have sexually offended (e.g., Aparcero et al., 2020; Kelley et al., 2018; McGrath et al., 2010), there has not been an updated practice survey of Canadian forensic sexual behavior programs since 2010. Practice surveys serve as a useful barometer of the state of applied practice and provide those working in these settings with an understanding of common practices. An updated practice survey of Canadian forensic sexual behavior programs is needed given several notable developments including, but not limited to, increased interest in utilizing secondary prevention services (e.g., Seto et al., 2024; Stephens et al., 2022), recent legal challenges and debates regarding the applicability of risk assessment tools to Indigenous clients (e.g., Olver et al., 2024), the development of new measures focused on protective factors for sexual offending (e.g., Nolan et al., 2023), and changes in legislation (e.g., the addition of non-consensual image distribution offenses to the Canadian Criminal Code). There have also been significant societal changes (e.g., the COVID-19 pandemic, increased attention to systemic inequalities) that would be expected to influence practice.

The present study expands upon and updates previous practice surveys. Our focus was on publicly funded Canadian forensic sexual behavior programs that provide services to individuals who have sexually offended or are at risk of doing so, with an emphasis on community programs. Although the focus is on Canada, this practice survey will likely be of broad interest to readers from other countries as it can highlight important similarities and differences in practice (McGrath et al., 2010). Further, mobility across jurisdictions suggests that practice across the Global North might parallel Canada, especially in that Canada has been a leader in advancements surrounding models of offender management (e.g., RNR) and risk assessment (e.g., most commonly used sexual recidivism risk tools were developed in Canada). Additionally, knowledge gained from practice surveys can be helpful in court when individuals are asked to defend certain practices or when program managers consider altering their programs. Lastly, results are informative for researchers to understand the uptake of research findings in applied settings.

## Method

### Sample and Procedure

We aimed to recruit all publicly funded community-based Canadian forensic sexual behavior programs that provide assessment and/or treatment to adults who have sexually offended or are at risk of offending. To be eligible to participate, programs must have been community-based and funded through public/government grants and contracts. Programs were identified through Internet searches, referrals from colleagues, government websites, and secondary referrals from initial points of contact. Eligible programs were invited via e-mail, which included information regarding the purpose of the study. The recruitment email explicitly mentioned that each program should only complete the survey once and encouraged email recipients to identify one staff member with the most insight into program practices to complete the survey. Programs that did not respond to the initial recruitment email were sent a follow-up email after two weeks, and a final recruitment email was sent four weeks after initial contact to inquire about participation.

A total of 23 community-based provincial sexual behavior programs were invited to participate, with 19 programs agreeing to participate (82.6% response rate). Of the 19 programs, three were excluded from the analyses as they did not respond to the entire survey. Thus, 16 programs provided information for the present study, with representation from all 10 Canadian provinces. Despite attempts to locate programs in the territories, it appeared that there were no community forensic sexual behavior programs in two of the territories (in a third, it appeared a pilot program might exist, but efforts to reach the program were unsuccessful). Specific characteristics of the 16 programs are detailed in Table 1.

**Table 1.** Program Characteristics (*N* = 16)

	*n*	%
Program location		
British Columbia	1	6.3
Alberta	3	18.8
Saskatchewan	1	6.3
Manitoba	1	6.3
Ontario	4	25.0
Quebec	2	12.5
New Brunswick	1	6.3
Nova Scotia	1	6.3
Prince Edward Island	1	6.3
Newfoundland and Labrador	1	6.3
Program setting (select all that apply)		
Community-based	14	87.5
Correctional	5	31.3
Residential	2	12.5
Number of clients (per year)		
0–50	5	31.3
51–100	3	18.8
101–200	2	12.5
201–300	5	31.3
301–500	1	6.3
Professions employed in program (select all that apply)		
Clinical psychologist	12	75.0
Social worker	10	62.5
Psychiatrist	7	43.8
Psychometrist/Psych technician	4	25.0
Counsellor/Psychotherapist	3	18.8
Occupational therapist	2	12.5
Nurse	2	12.5
Recreational therapist	1	6.3
Correctional staff	1	6.3
Behavioral therapist	1	6.3
Other	9	56.3

*Note.* For the question that asked about professionals employed, programs were able to list more than one in “other” category through open textboxes. “Other” included: co-therapists (*n* = 2), sexologists (*n* = 2), criminologist (*n* = 1), management (*n* = 1), police (*n* = 1), prosecution (*n* = 1), probation officers (*n* = 1), psychology students (*n* = 1), sexual offense specialists (*n* = 1), mental health/addictions workers (*n* = 1).

The initial plan was to solely focus on community-based forensic sexual behavior programs, but the research team subsequently extended the practice survey to correctional facilities within Canada that provide specialized sexual offending programming because they are also publicly funded. Seven provincial correctional institutions, and the body overseeing federal institutions, were identified as providing specialized programming. These institutions were contacted in the same manner as community-based programs. Unfortunately, contacting correctional-based forensic sexual behavior programs was associated with challenges and hurdles. Of the institutions that responded, it was noted that a lengthy application to conduct the research would be required. As a result, programs that operated solely within a correctional institution were not included in the present sample.

### Measures

An online survey was developed by the research team, which took participants an average of 29 minutes (*SD* = 18.0) to complete. The survey was divided into three sections (assessment, treatment, and prevention services). Skip logic was used so that sections could be skipped over if they were not relevant to the program (e.g., if a program stated they did not provide assessment services, they skipped this section of the survey). Survey questions were partially informed by the practice survey from McGrath et al. (2010); however, the present study expanded upon McGrath et al. (2010) in several ways. The survey covered six domains: (i) demographic and individual characteristics; (ii) staffing and training requirements; (iii) assessment practices; (iv) risk communication and report writing; (v) treatment procedures; and (vi) prevention approaches. The full survey is available in the supplemental material.

To explore individual characteristics of the programs, the survey asked specific demographic questions regarding the program’s location, years of operation, and professions employed in the program. Staffing and training requirements were investigated through survey questions regarding the number of staff employed within the program, requirements for staff to seek formal training on risk assessment tools, as well as questions regarding the specific responsibilities of each staff member (i.e., providing treatment, scoring risk instruments). To gather information regarding assessment practices, participants were asked about common measures used to assess personality or sexual preference, as well as risk tools used for specific populations (adult males, Indigenous clients, females, clients with child sexual exploitation material offenses (CSEM), clients with non-consensual image distribution charges (NCID), clients without a history of illegal sexual behavior). The next domain of the survey examined risk communication and report writing practices (e.g., total hours of client interview time). Treatment practices were explored through questions regarding the type of treatment intervention, group treatment targets, and risk level eligibility for treatment. Lastly, the survey asked about prevention services for clients who may be at risk of sexually offending (i.e., clients without a legal history) via two open-ended questions regarding their assessment and treatment practices when working with this population.

Following completion of the online survey, participants were provided with a feedback form and a link to enter a draw for a prize to win one of five $100 Amazon gift cards. Financial support for the present study was provided by Research Nova Scotia’s New Health Investigator Award (2021-1372). Ethics approval was granted by the authors’ university’s Research Ethics Board. The authors report no financial interests or conflicts of interest regarding the present research.

## Results

Descriptive information for three distinct areas of service delivery is provided (assessment, treatment, and prevention). The authors take responsibility for the integrity of the data and the accuracy of the analyses. Every effort has been made to avoid inflating the results.

### Risk Assessment Services

Most programs (81.3%, *n* = 13) provided comprehensive risk assessments and subsequently responded to a series of questions about their practices. Most of the 13 programs reported following a standardized risk assessment procedure for each client (76.9%, *n* = 10) and 61.5% (*n* = 8) provided clients with direct feedback about the assessment results. Additionally, 33.3% (*n* = 4)^1^ reported that they conduct file-based risk assessments only if a client does not provide their consent to participate in the assessment.

All programs that conducted risk assessments (*n* = 13) indicated that their assessment procedures included client interviews, collection of collateral information and a review of client files. Fewer programs, although still a majority, described use of actuarial (92.3%, *n* = 12) and structured professional judgement risk instruments (76.9%, *n* = 10), personality testing (61.5%, *n* = 8), and sexual preference testing (61.5%, *n* = 8).^1^ Fewer programs assessed response style, deception, and/or malingering (53.8%, *n* = 7) and cognitive testing (38.5%, *n* = 5).

The use of specialized sexual preference testing was reported in eight programs, and in the majority of these programs, phallometric testing was used (see Table 2). Additional questions were asked of programs that reported using phallometric testing (53.8%, *n* = 7). Of the programs that used phallometric testing, all utilized age/gender preference testing. Additionally, 85.7% (*n* = 6) provided sexual violence/coercion preference testing. Most programs indicated that their phallometric testing included both audio and visual stimuli (57.1%, *n* = 4), with fewer programs using only audio (28.6%, *n* = 2) or only visual stimuli (14.3%, *n* = 1). Most programs indicated that phallometric testing was used regardless of whether the sexual offense was committed against an adult or minor (71.4%, *n* = 5). Programs also described the process for determining which clients undergo phallometric testing and indicated that they consider factors such as client offense history (e.g., contact versus non-contact offense, age of victim/s), mental illness, client age and intelligence level, and whether testing was clinically indicated on the referral.

**Table 2.** Sexual Preference Testing Among Programs Providing Assessment (*N* = 13)

	*n*	%
Phallometric testing (*N* = 13)	7	53.8
Viewing time (*N* = 13)	4	30.8
Other cognitive measures of sexual interest (*N* = 12)	1	8.3
SSPI/SSPI-2 (*N* = 12)	3	25.0
Self-report questionnaires (*N* = 12)	6	50.0
Polygraph (*N* = 13)	-	-

*Note.* Dash (-) indicates zero participants selected this option. SSPI/SSPI-2 = Screening Scale for Pedophilic Interests/Revised Screening Scale for Pedophilic Interests.

Programs were also asked about their use of risk assessment tools. An average of 2.3 (*SD* = 1.0) risk assessment tools were used per assessment (Table 3 illustrates which risk tools were used when assessing different client populations). For adult male clients, the most common risk assessment tools were the Static-99/99R (Hanson & Thornton, 1999; Phenix et al., 2016; 84.6%, *n* = 11), followed by the STABLE-2007 (Hanson et al., 2007; 69.2%, *n* = 9), with the same being true for Indigenous adult male clients (Static-99/99R: 53.8%, *n* = 7; STABLE-2007: 53.8%, *n* = 7). Clients with CSEM offenses were most commonly assessed using the Child Pornography Offender Risk Tool (CPORT; Seto & Eke, 2015; 69.2%, *n* = 9) and STABLE-2007 (53.8%, *n* = 7). Similarly, the STABLE-2007 was the most used tool for clients with NCID offenses (38.5%, *n* = 5). Lastly, when assessing recidivism risk for adult female clients, four instruments were reported across the sample, with the most common being the Level of Service Inventory/Level of Service Inventory Revised (LSI/LSI-R; Andrews & Bonta, 1995; Andrews et al., 2004; 30.8%, *n* = 4). There was greater variation among risk assessment measures for those with a history of undetected sexual offenses. To explore assessment practices in greater depth, programs shared information regarding risk assessment report preparation and writing practices within their program (see Table 4).

**Table 3.** Frequencies of Risk Instruments Used Across Various Client Populations (Select all That Apply) (*n* = 13)

	Adult male		Indigenous male		CSEM only		NCID offenses		Adult female		Undetected	
	*n*	%	*n*	%	*n*	%	*n*	%	*n*	%	*n*	%
STATIC-99/99R	11	84.6	7	53.8	-	-	1	7.7	-	-	-	-
STABLE 2007	9	69.2	7	53.8	7	53.8	5	38.5	-	-	1	6.3
VRS-SO	5	38.5	5	38.5	3	23.1	-	-	-	-	2	12.5
PCL-R	5	38.5	4	30.8	2	15.4	3	23.1	2	15.4	2	12.5
SAPROF-SO	4	30.8	1	7.7	2	15.4	2	15.4	-	-	2	12.5
SVR-20	3	23.1	-	-	-	-	-	-	-	-	-	-
SAPROF	3	23.1	-	-	1	7.7	1	7.7	1	7.7	1	6.3
HCR-20	3	23.1	2	15.4	-	-	1	7.7	2	15.4	1	6.3
LSI/LSI-R	3	23.1	5	38.5	2	15.4	2	15.4	4	30.8	1	6.3
CPORT	3	23.1	1	7.7	9	69.2	-	-	-	-	-	-
STATIC-2002/2002R	2	15.4	1	7.7	-	-	-	-	-	-	-	-
VRAG-R	2	15.4	-	-	-	-	-	-	-	-	1	6.3
ACUTE 2007	2	15.4	1	7.7	1	7.7	-	-	-	-	-	-
SORAG	1	7.7	-	-	-	-	-	-	-	-	-	-
SOTIPS	1	7.7	1	7.7	1	7.7	-	-	-	-	-	-
VRAG	1	7.7	-	-	-	-	-	-	-	-	-	-
MnSOST-R	-	-	-	-	-	-	-	-	-	-	-	-
SRA	-	-	-	-	-	-	-	-	-	-	-	-
Other	5	38.5	2	15.4	1	7.7	1	7.7	-	-	1	6.3

*Note.* Dash (-) indicates zero participants selected this option. Of the 13 programs that provided risk assessments, one program did not respond to this question as they indicated being unaware of the specific tools used within their program for each population. NCID = Non-consensual intimate image distribution. VRS-SO = Violence Risk Scale Sexual Offense; PCL-R = Psychopathy Checklist Revised; SAPROF-SO = Structured Assessment of Protective Factors for Sexual Offending; SVR-20 = Sexual Violence Risk 20; SAPROF = Structured Assessment of Protective Factors for Violence Risk; HCR-20 = Historical Clinical Risk Management-20; VRAG-R = Violence Risk Appraisal Guide Revised; SORAG = Sex Offender Risk Appraisal Guide; SOTIPS = The Sex Offender Treatment Intervention and Progress Scale; MnSOST-R = Minnesota Sex Offender Screening Tool Revised; SRA = Structured Risk Assessment.

Programs were able to list more than one in “other” category through open textboxes. “Other” for adult male clients: VRS, ODARA (Ontario Domestic Assault Risk Assessment), RSVP (Risk for Sexual Violence Protocol), SAM-V2 (Stalking Assessment and Management Version 2), and ARMIDILO-S (Assessment of Risk and Manageability of Individuals with Developmental and Intellectual Limitations who Offend – Sexually).

“Other” for Indigenous adult males: VRS, ODARA, and RSVP.

“Other” for CSEM only/NCID/Undetected clients: RVSP.

**Table 4.** Risk Assessment Preparation and Report Writing Characteristics

	*n*	%
Time (hrs) to write report (*N* = 11)		
5 or less	3	27.3
6–10	6	54.5
11–15	-	-
16–20	1	9.1
21–25	-	-
26–30	1	9.1
31 or greater	-	-
Time (hrs) of client interaction (*N* = 12)		
Less than 3	-	-
3	3	25.0
4	3	25.0
5	2	16.7
6	1	8.3
8	1	8.3
10	1	8.3
15 or greater	1	8.3
Report length (pages; *N* = 12)		
10 or less	4	33.3
11–20	6	50.0
21–30	2	16.7
30 or greater	-	-

*Note.* Dash (−) indicates zero participants selected this option.

Some of the response options were collapsed in instances where no programs endorsed them.

### Treatment Services

All but one program reported offering treatment services (93.8%, *n* = 15). Over half of the programs offered virtual treatment services on a case-by-case basis (56.3%, *n* = 9), with fewer offering virtual treatment services to all clients (6.3%, *n* = 1), and the remainder not offering treatment in a virtual format (31.3%, *n* = 5). Of the 14 programs that responded to questions about monitoring treatment progress, three (21.4%) indicated that their clients completed pre-post questionnaires. In some programs, clinicians also scored risk assessment tools to assess treatment change (42.9%, *n* = 6).

Characteristics of the treatment approaches within the sample are noted in Table 5. Of the programs that provided treatment, all provided individual treatment (100.0%, *n* = 15), and most provided group (66.7%, *n* = 10, with 60% of these being closed groups) and maintenance programming (73.3%, *n* = 11). For group treatment, the average number of treatment sessions provided to a client was 32.0 sessions (*SD* = 23.9), and the average length of each session was 127.5 minutes (*SD* = 31.0). Six programs reported that they provide treatment to all risk levels (low to high risk; 40.0%, *n* = 6). Cognitive behavior therapy (CBT; 86.7%, *n* = 13) and Risk-Need-Responsivity (73.3%, *n* = 11) were the most commonly used treatment models, followed by the Good Lives Model (60.0%, *n* = 9). Emotional regulation (86.7%, *n* = 13), intimacy/relationship skills (86.7%, *n* = 13), offense supportive attitudes (80.0%, *n* = 12), and problem-solving skills (80.0%, *n* = 12) were the most common intervention targets found when asking participants to select the three most important targets during treatment.

**Table 5.** Characteristics of Treatment Approach (*N* = 15)

	*N*	%
Risk levels for treatment (select all that apply)		
Low	1	6.7
Low-moderate	6	40.0
Moderate	8	53.3
Moderate-high	6	40.0
High	6	40.0
All risk levels	6	40.0
Treatment models adhered to (select top 3)		
Cognitive behavioral therapy	13	86.7
Risk-need-responsivity	11	73.3
Good lives model	9	60.0
Psychosocial educational	5	33.3
Self-regulation	4	26.7
Relapse prevention	3	20.0
Harm reduction	2	13.3
Multisystemic	2	13.3
Sexual trauma	2	13.3
Sexual addiction	1	6.7
Biomedical	-	-
Family systems	-	-
Psychodynamic	-	-
Other	2	13.4
Group treatment targets (select all that apply)		
Emotional regulation	13	86.7
Intimacy/relationship skills	13	86.7
Offense supportive attitudes	12	80.0
Problem solving	12	80.0
Arousal control	11	73.3
Increasing personal supports	11	73.3
Social skills	11	73.3
Empathy	8	53.3
Offense responsibility	7	46.7
Other	2	13.3

*Note.* Programs were able to list more than one in “other” categories through open text boxes. “Other” group treatment targets were listed as: healthy sexuality, learning/identifying risk factors, victim impact, dynamic risk factors.

### Prevention Services

Prevention services were offered in 70.6% (*n* = 12) of programs, which involved service provision to those at risk of sexually offending and/or who do not have an official legal history. Of the programs that provided services to this population, half indicated that their program provided comprehensive risk assessments (50.0%, *n* = 6). In an open-ended textbox, participants were asked to elaborate on the risk assessment process used within their program for this population. Assessment practices varied, with only one of the six programs indicating that identical assessment services are provided to clients with and without a history of illegal sexual behavior (16.7%). Two programs indicated that their assessment practices included a clinical interview (33.3%), and one program reported the use of phallometric testing and mental health testing, if appropriate (16.7%). Other responses noted the use of only dynamic risk tools during assessments with this population (16.7%, *n* = 1), with another program indicating that criminogenic needs, sexual history, and problematic sexual behavior are assessed to guide treatment decisions (16.7%, *n* = 1). One program indicated that treatment eligibility for this population requires a referral from a professional and that self-referrals are not permitted (16.7%).

Among the 12 programs that provide prevention services, 75.0% (*n* = 9) reported that they provided treatment. Similar to the question described above, participants were provided an open textbox to describe treatment processes for this population. Three programs indicated that clients could be referred to individual or group treatment (33.3%), and two programs indicated that clients without a history of sexual offending receive the same treatment services as clients with legal convictions (22.2%). Other treatment measures detailed by participating programs included individual therapy/follow-up and risk management (22.2%, *n* = 2), pharmacological intervention (11.1%, *n* = 1), supportive counselling (11.1%, *n* = 1), and emotional regulation/sexual arousal management (11.1%, *n* = 1).

## Discussion

The results of our practice survey offer a useful overview of the assessment, treatment, and prevention services provided by publicly funded Canadian forensic sexual behavior programs. Our findings suggest that most programs provide assessment and treatment services, with fewer but still a notable majority providing prevention services. Most of the programs that conduct risk assessments follow a standardized process that, at a minimum, includes an interview with the client and collaterals as well as a review of file information. The majority of programs relied on actuarial risk instruments, most commonly using the Static-99R and STABLE-2007, and less so on structured professional judgement tools. The emphasis on actuarial measures is unsurprising based on previous research showing large effect sizes for actuarial risk assessment tools in predicting sexual recidivism (e.g., Hanson et al., 2009). Findings from the present study, in regard to risk assessment practices, are largely in line with the extant literature and best-practice guidelines (e.g., the use of the Static-99R; Bourgon et al., 2017; Kelley et al., 2018; McGrath et al., 2010).

The present study also highlights some notable differences from previous practice surveys, which likely reflect changes in the field that have occurred over time. These developments included the use of virtual services, the incorporation of newer measures into risk assessment (i.e., SAPROF-SO; Kelley et al., 2022), and the increase in the number of programs focusing on protective factors and holistic approaches to treatment (Subramonian & Severn, 2020). In terms of treatment practices, adherence to the Risk-Need-Responsivity model was more prevalent than in previous research (McGrath et al., 2010), with approximately 73.3% of programs indicating RNR to be a prominent approach within their treatment program. Similarly, our findings indicate a stronger adherence among Canadian forensic sexual behavior programs to the GLM compared to previous research (McGrath et al., 2010). Although the GLM is a more recent approach relative to the RNR model, support for strength-based rehabilitation frameworks such as the GLM continue to accumulate, alongside increasing implementation within forensic programs (Mallion et al., 2020; Ward, 2002; Zeccola et al., 2021). Nonetheless, empirical research on the efficacy of GLM-derived programming remains limited, partially due to the difficulty in distinguishing programs explicitly derived from the GLM from those that simply incorporate its principles into existing intervention models (Willis & Ward, 2024).

### Implications for Practice

Overall, results suggest that risk assessment practices are generally consistent with evidence-based practice. Most programs relied on a standardized approach and selected actuarial risk assessment tools that align with the current literature (e.g., Static-99R; Helmus et al., 2022; VRS-SO; Wielinga & Olver, 2024). Programs were flexible in their assessment approach based on client characteristics, which is consistent with the recommendation to ensure that the tools used are appropriate for the client group (e.g., Singh et al., 2014). The flexibility that programs demonstrated was generally consistent with the literature and recent advancements in the field. For example, Helmus et al. (2025) concluded that the CPORT and STABLE-2007 are defensible measures for assessing recidivism risk in men with CSEM offenses and we found that these were two commonly used tools with this population. In addition, most programs utilized just over two risk instruments per assessment and prioritized sexual recidivism risk tools over tools used for general or violent recidivism risk assessment, which suggests that programs may feel that the central focus of their assessment should be narrowly restricted to sexual recidivism risk. Further, programs included several other measures that would be expected to provide information for risk management implications, with the most prominent being personality assessment and sexual preference testing.

A notable development is the inclusion of assessment tools that incorporate protective factors, which can be viewed as an evolving area of practice with debates on the usefulness of this endeavor (e.g., Harris & Rice, 2015). Protective factors were considered in risk assessments in a sizeable number of programs, with 23.1% using the more general SAPROF to assess protective factors for violence risk in adult males and 30.8% using the SAPROF-SO, which is a recently developed measure that evaluates sexual offense specific protective factors (Kelley et al., 2022). Meta-analytic findings have generally found support for the association between the SAPROF and reduced risk of violent recidivism (*d* = .63); however, the effect size is smaller for sexual recidivism (*d* = .43), which is expected given that it is a broader violence protection tool that was not designed specifically for sexual violence (Burghart et al., 2023). The SAPROF-SO is still relatively new and not extensively validated, although initial findings suggest that higher levels of protection are associated with decreased sexual recidivism (Burghart et al., 2023; Carr et al., 2025). Overall, further research is needed on the value of assessing protective factors as part of a comprehensive risk assessment.

There was also evidence for adaptation in instances where no validated risk assessment tools were available for a particular population. For example, programs often relied on non-sexual recidivism risk assessment tools for assessing female clients, given the absence of a sexual offense specific risk assessment tool for this population (Cortoni, 2018). The absence of tools for this population is due to the low base rate of sexual recidivism in females, which introduces significant challenges to scale development (Cortoni et al., 2010). Although risk assessments are requested for the small subset of females who sexually offend and adaptation is necessary, there is still the potential for overestimation of risk and treatment needs when assessing female clients using general recidivism risk instruments (Russell & Darjee, 2013). Future research in this area, including the unique needs of females, is needed to help guide practice in this area.

The treatment results were more variable in their adherence to evidence-based practice, with some notable deviations from established findings. In regard to treatment programs, most programs reported adherence to evidence-based models, particularly CBT and RNR (Bonta & Andrews, 2017). The most frequently endorsed treatment targets were also consistent with research on dynamic risk factors (e.g., emotion regulation, relationship skills, and offense supportive attitudes; Helmus et al., 2013; Mann et al., 2010; Seto et al., 2024). On a less positive note, treatment practices also persist that deviate from evidence-based practice. For example, 40.0% of programs reported that they provide treatment to clients, regardless of risk level, suggesting a deviation from the risk principle. This was an interesting finding given that most programs reported adherence to RNR. Perhaps this suggests that programs may adhere to certain aspects of the model over others. Overall, programs should critically evaluate their adherence to RNR principles and make necessary changes, as greater adherence to RNR is associated with more sizable effects in reducing recidivism (e.g., Hanson et al., 2009).

In addition, treatment dosage appeared to deviate from recommendations within the extant literature, which can have unintended negative consequences of under/over-treating clients. In their white paper on offender assessment and treatment, Hanson et al. (2017) describe a five-level system of risk and needs where those classified as a Level I or Level II should not receive treatment and those classified as Level IIIs and Levels IVs should receive 100–200 and 200–300 hours of treatment, respectively. Conflicting with these recommendations, the present study found the average number of treatment sessions to be 32, with an average length of 127.5 minutes. A post hoc analysis indicated that the average group treatment dosage for the present study was 71.35 hours (ranged from 18 to 225 hours). Notably, the present study only asked programs about the duration of group treatment and therefore, additional treatment hours may have been provided through individual treatment sessions. Furthermore, the majority of programs surveyed in the present study were offered in the community, where offering 100-300 hours of treatment could pose logistical challenges. Nonetheless, treatment dosage guidelines have not been the focus of extensive research and require further validation (McGrath et al., 2010).

Lastly, it was intriguing that 70.6% of programs reported that they provide prevention services. The majority of programs that provided prevention services were focused on treatment followed by assessment. It appears that programs have taken an approach that involves incorporating these clients into their standard clinical offerings, whereas others have developed specialized programs for these individuals that differ from their standard clinical practice. The delivery of secondary prevention services is still in its infancy, with evidence-based practices yet to be firmly established (e.g., Stephens et al., 2022).

### Additional Considerations for the Implementation of Research into Practice

Our findings indicate potential gaps between research and the implementation of findings into practice. Previous research has suggested that it takes approximately 17 years after discovery to implement findings into clinical practice (Lenfant, 2003; Morris et al., 2011). Although this is a general estimate, it highlights the important point that there is a delay in implementation, which may explain why certain domains of service were reported less frequently in previous practice surveys (e.g., McGrath et al., 2010) than in the present study.

Despite this well-documented time-lag between research and practice, there are some practices that seem to be implemented more quickly than others, which may reflect greater intricacies than a fixed time lag. For example, the provision of virtual mental health services has increased substantially and has witnessed significant advancement over the 21^st^ century (e.g., Lal & Adair, 2014); however, the use of technology in the forensic sector has remained stagnant, underdeveloped, and underutilized (Kois et al., 2021). Nonetheless, during the COVID-19 pandemic, virtual services were quickly adopted out of necessity, regardless of the absence of formal guidelines and regulations (Kois et al., 2021). Furthermore, the use of the GLM and emphasis on protective factors has increased in popularity, despite having less empirical support than other methodologies (e.g., Zeccola et al., 2021). Perhaps this relatively rapid implementation reflects a broader bias toward considering individuals’ positive attributes, aligning with the growing influence of the positive psychology movement (de Vries Robbé & Willis, 2017).

In contrast, many older practices continue to be used that are not empirically supported, suggesting difficulty in adopting newer methodologies. For example, 40.0% of surveyed programs in the present study reportedly provide treatment to clients of all recidivism risk levels, despite widespread understanding that providing treatment to those who are low risk likely does more harm than good (e.g., Hanson et al., 2017). Given the lack of empirical support for certain practices, it is curious why certain approaches persist. Of note, Cook et al. (2009) surveyed 1600 psychotherapists to understand the barriers to adopting new treatments and found that clinician attitudes, client characteristics, contextual/institutional factors, and training issues contribute to delays in the use of empirically validated treatments. Specifically, a lack of clinician confidence, limited time and finances to complete training, and a lack of opportunities to refine treatment skills were commonly cited as factors that contribute to an unwillingness to engage in new treatment techniques (Cook et al., 2009). In a similar vein, researchers in the health services field have suggested that while the delay between knowledge acquisition and implementation is long, the delay that exists for de-adopting harmful and/or unnecessary practices may reflect an even larger gap (Niven et al., 2015).

In short, various factors may influence the quick implementation of new treatments and risk tools, and others may contribute to delays in uptake. Although there is evidence of certain practices being implemented prior to the accumulation of ample empirical support, it appears as though these instances reflect a deviation from the norm rather than the norm itself. Rather, most newly implemented practices appear to take a considerable amount of time to implement and may take even longer to de-adopt once empirical support has dwindled.

### Strengths and Limitations

Our study has several strengths, including strong representation across all ten Canadian provinces, a comprehensive overview of a wide range of practices, and a high response rate. Nonetheless, the results should be interpreted with several limitations in mind. Although the research team originally did not plan on including specialized correctional-based sexual behavior programs, we later extended the survey to include these programs because they are also publicly funded. There were challenges recruiting specialized correctional programs as they often required additional research clearance for program staff to participate, which was not feasible in the current project. Further, it is possible that we missed programs that were relatively new or lacked a strong online presence. We attempted to offset this limitation by asking participating programs to share the contact information of other programs that would be eligible for inclusion. Lastly, the survey was only available in English, which may have precluded participation from some programs that were offered exclusively in French if there was not a bilingual staff member willing to complete the survey. However, this limitation was somewhat offset by our ability to recruit programs in Quebec (*n* = 2).

### Future Research Directions

The present study highlights several fruitful avenues for future research. Future research should further explore the implementation of research into practice and barriers to providing evidence-based clinical services in forensic sexual behavior programs. For instance, it may be beneficial to explore how certain treatment models are translated into practice within forensic sexual behavior programs. Additionally, it would be useful to continue to conduct the same practice survey every few years to monitor changes in practice over time. Future research could also extend the current findings by including private practices and other countries, especially in the Global South (e.g., Aparcero et al., 2020; Singh et al., 2014). Lastly, researchers should conduct research aimed at better understanding the factors that lead to research recommendations for forensic practice and barriers to the implementation of evidence-based practice.

## Supplemental Material

Supplemental Material - A Practice Survey of Canadian Forensic Sexual Behavior Programs Supplemental Material for A Practice Survey of Canadian Forensic Sexual Behavior Programs by Payton McPhee, Jordyn Monaghan, and Skye Stephens in Sexual Abuse.

## Note

1. Out of the 13 programs that provided risk assessments, one program did not respond to this question, and therefore, the total *N* was revised to reflect the missing data (*N* = 12).
