# Supplementary material for: A Practice Survey of Canadian Forensic Sexual Behavior Programs
Source: Sex Abuse. 2025 Sep 11;38(3):354–75. doi: 10.1177/10790632251377706 (PMC12916884; doi:10.1177/10790632251377706)
Supplement: Supplemental Material - A Practice Survey of Canadian Forensic Sexual Behavior Programs [file sj-pdf-1-sax-10.1177_10790632251377706.pdf]

Do you consent to participate in this study?

- ○ I consent
- ○ I do not consent

Does your program work with adults (18+) who have sexually offended (either detected or undetected) or are at risk of doing so?

- ○ No
- ○ Yes

The purpose of the present survey is to better understand current practices in Canadian programs for those who have sexually offended or are at risk of doing so. When completing the following survey please only consider the secondary prevention services (services to at-risk populations, such as those with paraphilic interests that could bring them into contact with the law) AND/OR tertiary prevention services (to those who have sexually offended) that you provide to adults (18+).

What is the name of your program?

____

What is your profession within this program?

- ○ Clinical Psychologist
- ○ Social Worker
- ○ Behavioral Therapist
- ○ Counsellor/Psychotherapist
- ○ Psychometrist/Psychology Technician
- ○ Psychiatrist
- ○ Occupational Therapist
- ○ Recreational Therapist
- ○ Nurse
- ○ Correctional Staff (probation officer, security, etc.)
- ○ Other (please explain below) ____
- ○ I do not currently work at this program

How long have you worked at the program?

- ○ Less than 1 year
- ○ 1 - 5 years
- ○ 6 - 10 years
- ○ 11 - 15 years
- ○ 16 - 20 years
- ○ More than 20 years

What are the different professions that work in your program [Check all that apply]?

- ☐ Clinical Psychologist
- ☐ Social Worker
- ☐ Behavioral Therapist
- ☐ Counsellor/Psychotherapist
- ☐ Psychometrist/Psychology Technician
- ☐ Psychiatrist
- ☐ Occupational Therapist
- ☐ Recreational Therapist
- ☐ Nurse
- ☐ Correctional Staff (probation officer, security, etc.)
- ☐ Other (please explain below) ____

Where is your program physically located?

- ▢ Alberta
- ▢ British Columbia
- ▢ Quebec
- ▢ Manitoba
- ▢ New Brunswick
- ▢ Newfoundland and Labrador
- ▢ Northwest Territories
- ▢ Nova Scotia
- ▢ Nunavut
- ▢ Ontario
- ▢ Prince Edward Island
- ▢ Saskatchewan
- ▢ Yukon Territory
- ▢ The program is not located in Canada

What type of services does the program provide [Check all that apply]?

- ▢ Community-based services
- ▢ Correctional services
- ▢ Residential or inpatient services
- ▢ Other (please explain below) ____

How many years has the program been running?

▼ Less than 1 ... 20+

Please provide an estimate of the average number of clients that the program serves each year.

- ○ 0 - 50
- ○ 51 - 100
- ○ 101 - 200
- ○ 201 - 300
- ○ 301 - 500
- ○ 501 - 800
- ○ 801 - 1000
- ○ >1000

How is your program funded [Check all that apply]?

- ☐ Private, for profit
- ☐ Private, not for profit
- ☐ Public
- ☐ Private-public partnership
- ☐ Research grants and/or research contract
- ☐ Other (please explain below) ____

Approximately how many staff work at the program?

- ○ 1 person
- ○ 2 - 5 people
- ○ 6 - 10 people
- ○ 11 - 20 people
- ○ More than 20 people

Do you require staff to seek certified training on specific risk assessment tools?

- ○ No
- ○ Yes

The following questions ask you to consider what professionals are responsible for the following tasks. Please select all that apply.

Providing treatment (individual and/or group):

- ▢ Registered Psychologist
- ▢ Registered Social Worker
- ▢ Psychiatrist
- ▢ Correctional Staff (probation officer, security, etc.)
- ▢ Psychometrist/Psychology Assistant/Psychology Technician If yes, please specify education level required (i.e., BA/BSc in psychology, MSW, etc.) ____
- ▢ Behavior Technician If yes, please specify education level required (i.e., BA/BSc in psychology, MSW, etc.) ____
- ▢ Registered Counsellor
- ▢ Registered Occupational Therapist
- ▢ Recreational Therapist
- ▢ Registered Nurse
- ▢ Licensed Practical Nurse
- ▢ Other (please explain below) ____
- ▢ Not applicable - my program does not provide treatment

Administrating psychological measures:

- ▢ Registered Psychologist
- ▢ Registered Social Worker
- ▢ Psychiatrist
- ▢ Correctional Staff (probation officer, security, etc.)
- ▢ Psychometrist/Psychology Assistant/Psychology Technician If yes, please specify education level required (i.e., BA/BSc in psychology, MSW, etc.) ____
- ▢ Behavior Technician If yes, please specify education level required (i.e., BA/BSc in psychology, MSW, etc.) ____
- ▢ Registered Counsellor
- ▢ Registered Occupational Therapist
- ▢ Recreational Therapist
- ▢ Registered Nurse
- ▢ Licensed Practical Nurse
- ▢ Other (please explain below) ____

- ☐ Not applicable - my program does not administer psychological measures

Scoring and interpreting risk assessment tools:

- ☐ Registered Psychologist
- ☐ Registered Social Worker
- ☐ Psychiatrist
- ☐ Correctional Staff (probation officer, security, etc.)
- ☐ Psychometrist/Psychology Assistant/Psychology Technician If yes, please specify education level required (i.e., BA/BSc in psychology, MSW, etc.) ____
- ☐ Behavior Technician If yes, please specify education level required (i.e., BA/BSc in psychology, MSW, etc.) ____
- ☐ Registered Counsellor
- ☐ Registered Occupational Therapist
- ☐ Recreational Therapist
- ☐ Registered Nurse
- ☐ Licensed Practical Nurse
- ☐ Other (please explain below) ____
- ☐ Not applicable - my program does not score and interpret risk assessment tools

Writing the assessment report:

- ▢ Registered Psychologist
- ▢ Registered Social Worker
- ▢ Psychiatrist
- ▢ Correctional Staff (probation officer, security, etc.)
- ▢ Psychometrist/Psychology Assistant/Psychology Technician If yes, please specify education level required (i.e., BA/BSc in psychology, MSW, etc.) ____
- ▢ Behavior Technician If yes, please specify education level required (i.e., BA/BSc in psychology, MSW, etc.) ____
- ▢ Registered Counsellor
- ▢ Registered Occupational Therapist
- ▢ Recreational Therapist
- ▢ Registered Nurse
- ▢ Licensed Practical Nurse
- ▢ Other (please explain below) ____
- ▢ Not applicable - my program does not write assessment reports

The next several questions will ask you about assessment practices with those who have been detected for sexual offending, unless otherwise specified. If your program does not provide assessment, there will be an option to skip over this section.

Does the program you work for provide risk assessments for people who have sexually offended (either detected or undetected)?

- ○ No
- ○ Yes

Does your program have a standardized assessment process for each client?

- ○ No
- ○ Yes

If a client does not consent to the assessment, do you provide file-based risk assessments?

- ○ No
- ○ Yes

Typically, what is involved in your risk assessment process:

Interview with client

- ○ No
- ○ Yes

Pen and paper personality testing (e.g., PAI)

- ○ No
- ○ Yes

Cognitive testing (e.g., WAIS)

- ○ No
- ○ Yes

Assessment of response style, deception, and/or malingering

- ○ No
- ○ Yes

Collecting collateral information via file review or interview

- ○ No
- ○ Yes

General file review

- ○ No
- ○ Yes

Actuarial risk assessment instruments

- ○ No
- ○ Yes

Structured professional judgement risk instruments

- ○ No
- ○ Yes

Sexual preference testing

- ○ No
- ○ Yes

Please describe any other practices or procedures which may be involved in your risk assessments

____

In the table below, we have indicated specific populations that programs may provide clinical services to. Populations included in the table below include: a) adult men; b) adult Indigenous men; c) adult females; d) individuals who have been charged with a child sexual exploitation material offence and have no history/charges of contact offending; e) individuals who have been charged with nonconsensual image distribution (other than CSEM offences); and f) those who have no history of detected sexual offending. Please indicate which risk instruments your program would typically use when providing assessments to each population.

	Adult Male	Adult Indigenous Male	Adult Female	Individuals with Child Sexual Exploitation (CSEM) offences only (no history of contact offending)	Individuals with Nonconsensual Image Distribution offences (does not include CSEM)	Undetected Offending (no sexual offence resulting in charge or conviction)
Static 99/99R	☐	☐	☐	☐	☐	☐
Static 2002/2002R	☐	☐	☐	☐	☐	☐
Stable 2007	☐	☐	☐	☐	☐	☐
SORAG	☐	☐	☐	☐	☐	☐
VRAG-R	☐	☐	☐	☐	☐	☐
SVR-20 (any version)	☐	☐	☐	☐	☐	☐
CPORT	☐	☐	☐	☐	☐	☐
MNSOTR	☐	☐	☐	☐	☐	☐
SRA - Structured Risk Assessment	☐	☐	☐	☐	☐	☐
SOTIPS	☐	☐	☐	☐	☐	☐
VRS-SO	☐	☐	☐	☐	☐	☐
Acute-2007	☐	☐	☐	☐	☐	☐
SAPROF-SO	☐	☐	☐	☐	☐	☐
SAPROF	☐	☐	☐	☐	☐	☐
VRAG	☐	☐	☐	☐	☐	☐

HCR-20 (regardless of version)	☐	☐	☐	☐	☐	☐
LSI/LSI-R	☐	☐	☐	☐	☐	☐
PCL-R	☐	☐	☐	☐	☐	☐
Other: Please write any other tools used and the population they are used with in the box below	☐	☐	☐	☐	☐	☐

Generally, how many risk assessment tools do you use per risk assessment?

▼ 1 ... 10 or more

What personality measures does your program typically administer?

- ○ MMPI-2
- ○ MMPI-2 RF
- ○ MMPI-3
- ○ MCMI-3
- ○ MCMI-4
- ○ PAI
- ○ The program does not administer personality measures
- ○ Other (please explain below) ____

Does your program use any of the following sexual preference measures:

Phallometric Testing

- ○ No
- ○ Yes

Viewing Time

- ○ No
- ○ Yes

Other cognitive measures of sexual interest (e.g., Implicit Association Test for sexual preference)

- ○ No
- ○ Yes

Polygraph

- ○ No
- ○ Yes

SSPI/SSPI-2

- ○ No
- ○ Yes

Self-Report Questionnaires (e.g., Multiphasic Sex Inventory)

- ○ No
- ○ Yes

Please list any other sexual preferences measures used by your program that were not listed above

____

How do you determine which clients are required to participate in phallometric testing?

____

Does your program administer the following type of phallometric tests?

- ☐ Age/gender preference test (to see the age and gender people are attracted to)
- ☐ Sexual violence/coercion/sadism preference
- ☐ Neither
- ☐ Other ____

Does your program only administer age/gender preference test to those with known offences against children?

- ○ No
- ○ Yes

Phallometric testing modality?

- ○ Audio
- ○ Visual
- ○ Both
- ○ Other ____

How many hours do you estimate it takes staff to write a typical risk assessment report?

▼ Less than 5 ... More than 80

For a standard risk assessment, what is the average amount of hours of total client interaction time?

▼ 0 ... 15 +

Generally, how many pages is the average risk assessment report in your program?

- ○ 10 pages of less
- ○ 11 - 20 pages
- ○ 21 - 30 pages
- ○ 31 - 40 pages
- ○ 41 - 50 pages
- ○ More than 50 pages

Does your program provide dangerous offender/long term offender assessments?

- ○ No
- ○ Yes

Does your program typically provide direct feedback to clients about their assessment results?

- ○ No
- ○ Yes

The next set of questions ask about treatment practices with those who have been detected for sexual offending. If your program does not provide treatment, there will be an option to skip over this section.

Does the program you work for provide treatment for people who have sexually offended (either detected or undetected)?

- ○ No
- ○ Yes

What types of treatment interventions does your program provide [Check all that apply]?

- ☐ Individual Treatment
- ☐ Group Therapy
- ☐ Maintenance Programming (individual and/or group)
- ☐ Other ____

Please select the primary 3 treatment models that best describe the program's approach [Please select 3 options]?

- ☐ Good Lives Model
- ☐ Cognitive Behavioral Therapy
- ☐ Relapse Prevention
- ☐ Risk Needs Responsivity

- ▢ Bio medical
- ▢ Family systems
- ▢ Harm reduction
- ▢ Multisystemic
- ▢ Psychodynamic
- ▢ Psychosocial educational
- ▢ Self-regulation
- ▢ Sexual addiction
- ▢ Sexual trauma
- ▢ Other ____

Does your program offer virtual therapy services?

- ○ No
- ○ Yes, all treatment services are offered virtually
- ○ Yes but only some of our treatment services are offered virtually or on a case by case basis (e.g., client unable to attend due to distance)

If your program offers group therapy, what type of groups do you provide?

- ○ Open
- ○ Closed
- ○ My program does not offer group treatment

What are the group treatment targets [Check all that apply]?

- ▢ Offence supportive attitudes
- ▢ Arousal control/sexual self-regulation
- ▢ Emotional regulation/coping with emotions
- ▢ Increasing personal supports
- ▢ Intimacy/ relationship skills
- ▢ Offense responsibility
- ▢ Problem solving
- ▢ Social skills
- ▢ Empathy
- ▢ Other ____

Dosage of the Group Treatment Program:

Number of sessions on average?

____

Length of each session (in minutes)?

____

How often are sessions provided?

- ○ Daily
- ○ Multiple times per week
- ○ Weekly
- ○ Biweekly
- ○ Monthly
- ○ Other ____

How does your program determine who is and who is not provided with treatment?

____

What risk level does the treatment program target [Check all that apply]?

- ☐ Low
- ☐ Low-moderate
- ☐ Moderate
- ☐ Moderate-high
- ☐ High
- ☐ All risk levels

Do clients complete paper and pencil measures of treatment change?

- ○ No
- ○ Yes

Does your program routinely have clinicians score risk instruments to assess treatment change?

- ○ No
- ○ Yes

The last few questions ask about practices for those who may be at risk of sexual offending but have no official legal history. In other words, we would like to ask you a few questions about secondary

prevention services you may provide. If your program does not provide these services, there will be an option to skip over this section.

Does your program provide services to those who do not have an official legal history but are concerned about their risk of offending AND/OR those with atypical sexual interests that may enhance their risk of sexual offending (e.g., clients with pedophilic interests)

- ○ No
- ○ Yes

Do you provide comprehensive assessments to those who do not have an official legal history but are concerned about their risk of offending AND/OR those with atypical sexual interests that may enhance their risk of sexual offending (e.g., clients with pedophilic interests)

- ○ No
- ○ Yes

Please provide a brief description of the assessment services that are provided

____

Do you provide treatment to those who do not have an official legal history but are concerned about their risk of offending AND/OR those with atypical sexual interests that may enhance their risk of sexual offending (e.g., clients with pedophilic interests)

- ○ No
- ○ Yes

Please provide a brief description of the treatment services that are provided

____

Is there anything about your program that we did not ask about that you would like us to know?

____

Are there any other specialized services for those who have sexually offended (secondary or tertiary prevention) in your jurisdiction that you think we should contact to fill out the survey? If yes, please provide the specific name of the program you think we should contact.

- ○ No
- ○ Yes (please specify name of program)

____

Would you like a copy of the results of the survey? If yes, please provide an email address.

- ○ No
- ○ Yes (enter e-mail) ____

Would you like to enter the draw for a chance to win 1 of 5 $100 Amazon gift cards to purchase books for your program? If yes, please provide an email.

- ○ No
- ○ Yes (enter e-mail) ____
